# Virus–Host Interactions Between Nonsecretors and Human Norovirus

**DOI:** 10.1016/j.jcmgh.2020.03.006

**Published:** 2020-04-11

**Authors:** Lisa C. Lindesmith, Paul D. Brewer-Jensen, Michael L. Mallory, Kara Jensen, Boyd L. Yount, Veronica Costantini, Matthew H. Collins, Caitlin E. Edwards, Timothy P. Sheahan, Jan Vinjé, Ralph S. Baric

**Affiliations:** 1 Department of Epidemiology, University of North Carolina, Chapel Hill, North Carolina; 2 Division of Viral Diseases, Centers for Disease Control and Prevention, Atlanta, Georgia; 3 Hope Clinic of the Emory Vaccine Center, Division of Infectious Diseases, Department of Medicine, School of Medicine, Emory University, Decatur, Georgia

**Keywords:** Neutralizing Antibody, Blockade Antibody, Bile, Receptor Binding, Cellular Immunity, cDNA, complementary DNA, GCDCA, glycochenodeoxycholic acid, HBGA, histoblood group antigen, HIE, human intestinal enteroid, IFN, interferon, IL, interleukin, NK, natural killer, PBMC, peripheral blood monocytic cell, PCR, polymerase chain reaction, PGM, pig gastric mucin, TCA, taurocholic acid, Th, helper T cell, TNF, tumor necrosis factor, VLP, virus-like particle

## Abstract

**Background & Aims:**

Human norovirus infection is the leading cause of acute gastroenteritis. Genetic polymorphisms, mediated by the *FUT2* gene (secretor enzyme), define strain susceptibility. Secretors express a diverse set of fucosylated histoblood group antigen carbohydrates (HBGA) on mucosal cells; nonsecretors (*FUT2*^-/-^) express a limited array of HBGAs. Thus, nonsecretors have less diverse norovirus strain infections, including resistance to the epidemiologically dominant GII.4 strains. Because future human norovirus vaccines will comprise GII.4 antigen and because secretor phenotype impacts GII.4 infection and immunity, nonsecretors may mimic young children immunologically in response to GII.4 vaccination, providing a needed model to study cross-protection in the context of limited pre-exposure.

**Methods:**

By using specimens collected from the first characterized nonsecretor cohort naturally infected with GII.2 human norovirus, we evaluated the breadth of serologic immunity by surrogate neutralization assays, and cellular activation and cytokine production by flow cytometry.

**Results:**

GII.2 infection resulted in broad antibody and cellular immunity activation that persisted for at least 30 days for T cells, monocytes, and dendritic cells, and for 180 days for blocking antibody. Multiple cellular lineages expressing interferon-γ and tumor necrosis factor-α dominated the response. Both T-cell and B-cell responses were cross-reactive with other GII strains, but not GI strains. To promote entry mechanisms, inclusion of bile acids was essential for GII.2 binding to nonsecretor HBGAs.

**Conclusions:**

These data support development of within-genogroup, cross-reactive antibody and T-cell immunity, key outcomes that may provide the foundation for eliciting broad immune responses after GII.4 vaccination in individuals with limited GII.4 immunity, including young children.

## Graphical abstract

**Figure:**
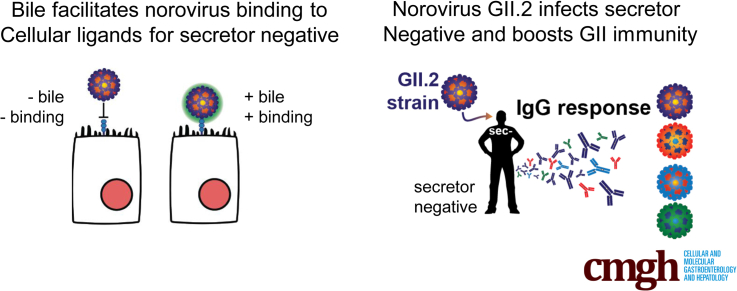

**Summary**

Nonsecretors of histoblood group antigens are genetically resistant to many human norovirus strains owing to a lack of available receptors. GII.2 strain binding to nonsecretor entry ligands is facilitated by bile. Upon GII.2 infection, cross-genotype immune responses are boosted.

Human norovirus infection is the leading cause of acute gastroenteritis worldwide, estimated to cause >220,000 deaths per year, mostly in children younger than 5 years old in developing countries.^1^ The high disease burden is facilitated by the large number of antigenically distinct strains, frequent recombination between strains, and the rapid evolution of surface epitopes in the pandemic GII.4 human norovirus strains, complex pre-exposure histories, and host genetics.^2^, ^3^, ^4^, ^5^, ^6^ Serial pandemics of human norovirus occurred in 1995, 2002, 2004, 2006, 2009, and 2012, fueled by virus evolution and antigenic drift within the predominant GII.4 strains that rendered herd immunity ineffective.^5^^,^^7^, ^8^, ^9^ The years between GII.4 pandemics typically are characterized by an increased prevalence of non-GII.4 strains including GII.17 in 2014–2015, and GII.2 in 2016–2017.^10^

Host genetic variation and pre-exposure histories influence pandemic outcomes and the spread of virus through the human population. Histoblood group antigens (HBGAs), commonly known as blood type antigens when expressed on red blood cells, are essential factors for human norovirus cell docking, reviewed by Nordgren and Svensson.^11^ The *FUT2* gene encodes an α-1,2-fucosyltransferase, the secretor enzyme, which adds a fucose to the H antigen precursor, generating H antigen, which is expressed on the surface of mucosal cells and secreted into mucosal fluids, a secretor phenotype. A wide variety of HBGAs are derived from the H antigen. Inactivating mutations in the secretor enzyme yield a nonsecretor phenotype and expression of a limited set of HBGAs on mucosal cells and in mucosal secretions. Secretor phenotypes trend geographically, ranging from >70% secretors in Central America to >70% nonsecretors in parts of Southeast Asia.^12^ Regardless of secretor type, HBGAs can be modified by addition of α1-3,4-fucose by the Lewis enzyme encoded by *FUT3* generating Lewis b in mucosal secretions of secretors and Lewis a in nonsecretors. A and B enzymes may add additional sugar moieties to the H antigen, further diversifying the HBGA pool available for virus interaction.

Both secretor and Lewis phenotypes are associated with human norovirus susceptibility.^11^^,^^13^ For example, GI.1 infection is restricted to secretor-positive populations^13^; GII.4 infection is restricted primarily to secretor-positive populations^14^^,^^15^; and GII.3, GII.7, and GII.6 infection is secretor-independent, infecting both secretors and nonsecretors.^15^, ^16^, ^17^, ^18^, ^19^, ^20^ Consequently, human norovirus–HBGA interaction is strain-dependent. Pandemic GII.4 strains typically bind to a diverse selection of secretor HBGAs and infect secretors of all blood types. Notably, select pandemic strains bind nonsecretor HBGAs in vitro and infect nonsecretors.^5^^,^^21^ GII.4 binding diversity is facilitated by microvariation in residues surrounding the HBGA binding pocket that stabilize secondary contacts with sugar moieties outside the fucose primary contacts.^5^^,^^22^^,^^23^ Along with antigenic change, broad docking-ligand use contributes to the global dominance of the GII.4 strains.

In contrast, GII.2 virus-like particles (VLPs) do not bind to any tested synthetic carbohydrates or the multivalent natural carbohydrate pig gastric mucin that comprises several secretor HBGAs.^16^^,^^24^^,^^25^ GII.2 VLPs do bind to human type B saliva. This in vitro binding pattern is incongruous with the in vivo infection model for GII.2 strains because secretors, blood types O, A, and B, and 1 nonsecretor have been infected experimentally with high-dose GII.2 Snow Mountain virus.^16^ Recently, high-resolution cryoelectron microscopy of a GII.2 VLP described the capsid surface loops involved in binding of HBGAs in the ligand binding pocket as highly flexible. Asp383, a conserved amino acid within the HBGA binding pocket that directly interacts with the fucose moiety of HBGAs, can be rotated toward or away from the HBGA binding site, potentially accounting for lack of GII.2 binding under most conditions, although the stimuli needed for rotameric shifts are unknown.^26^ However, Jung et al further describe zinc ion binding near the HBGA binding loops and speculate the ion may be involved with stabilizing the loops, facilitating HBGA binding. Similar interactions were reported for GII.1 human norovirus VLPs and mouse norovirus,^27^^,^^28^ indicating that a diverse spectra of environmental factors may modulate norovirus cell attachment and infectivity.

Similar to influenza A, human norovirus strain exposure history shapes immunity after infection and vaccination.^29^, ^30^, ^31^ In adults, soon after vaccination and infection, antibodies able to block HBGA ligand binding of multiple strains in a surrogate neutralization assay are detected in serum, indicating common epitopes and potential targets for vaccine-induced broad protection.^31^, ^32^, ^33^, ^34^ Importantly, blockade antibodies correlate with protection from infection and neutralization of virus in vitro.^35^, ^36^, ^37^ Multiple exposures likely are needed to induce adequate cross-genotype neutralizing antibody responses because very young children and some adults frequently experience repeat infection of strains within the same genogroup.^35^^,^^38^, ^39^, ^40^ Non–antibody-mediated immune responses to human norovirus infection largely are undefined beyond interferon (IFN)-ɣ and interleukin (IL)2 detection in serum and fecal samples after infection and cellular ex vivo stimulation with virus capsid.^16^^,^^33^^,^^41^ Nonsecretors experience a restricted range of human norovirus infections compared with secretors, however, the impact of this reduced immunologic exposure on antibody and cellular immune responses, vaccine outcomes, and susceptibility to emergent strains remains unknown, hampering our ability to predict and evaluate vaccine performance in this population.

Understanding the balance between host-mediated susceptibility and immunity and virus-mediated diversity and evolution will be critical in understanding norovirus cross-strain immunity to inform vaccine design and performance. Here, we characterize both serologic and cellular immunity after natural GII.2 infection in a familial nonsecretor cohort defining the breadth of immune responses across human norovirus strains in individuals with limited GII.4-driven immunity. Furthermore, we define additional co-factors needed for GII.2 binding to nonsecretor HBGAs. These data provide a mechanistic explanation for secretor and nonsecretor genetic susceptibility to GII.2 infection, provide key GII.2 baseline data for human challenge and vaccine studies in nonsecretor populations, and provide novel models to evaluate the serologic basis for cross-protective immune responses in GII-infected nonsecretors after infection or vaccination.

## Results

### GII.2 Human Norovirus Infects Nonsecretors

**Figure 1:**
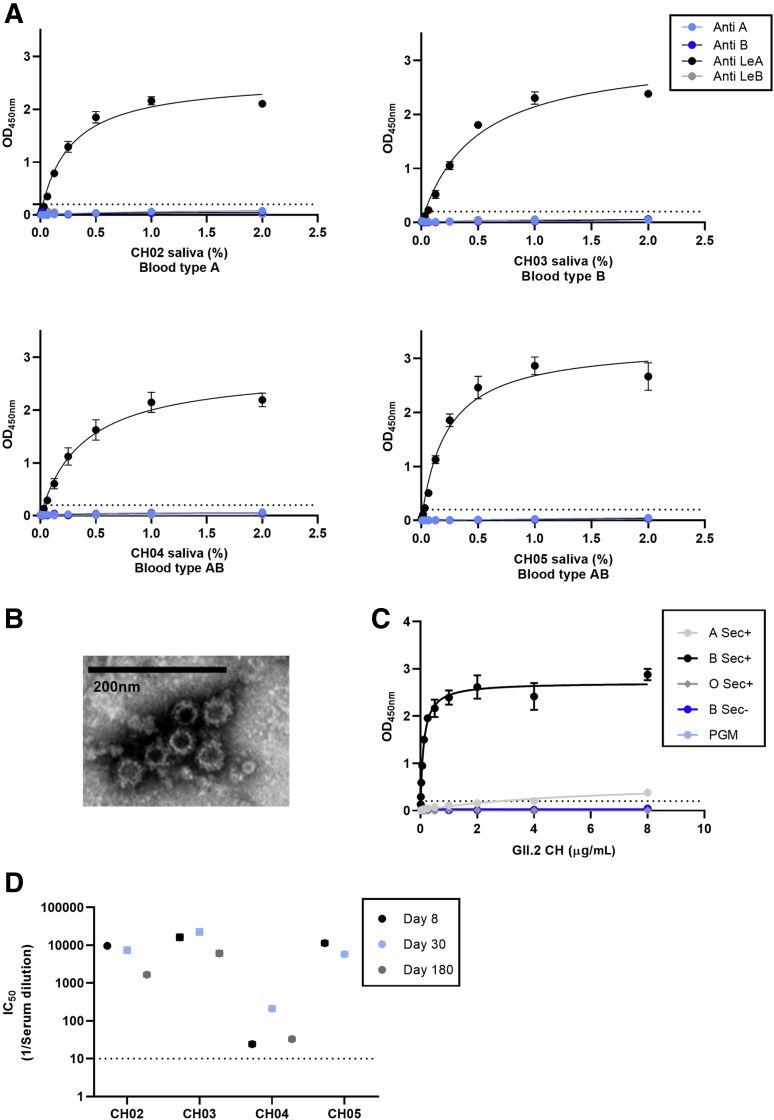
**Human norovirus GII.2 CH outbreak donor and strain characterization.** (*A*) Donors are Lewis-positive, nonsecretors based on salivary secretion of Lewis a in the absence of H, Lewis b, A or B HBGA. (*B*) A GII.2 human norovirus strain was extracted from a stool sample collected 2 days after symptom onset and the GII.2 CH capsid gene was sequenced and expressed as VLPs. Original magnification: ×100,000. (*C*) GII.2 CH VLP-bound human type B saliva but not PGM, as reported for other GII.2 VLPs. (*D*) Donors had high ligand-binding blockade antibody titers to GII.2 CH at day 8 that persisted until the end of sample collection (1–6 mo), supporting GII.2 infection in all 4 donors. Markers denote the (*A* and *C*) means and SEM or (*D*) 95% CIs from a minimum of 2 replicates tested in 2 independent experiments. The *dashed line* is the lower limit of detection. IC_50_, 50% inhibitory concentration; Sec, secretor.

In November 2017, a family cohort, designated Chapel Hill outbreak (CH), experienced acute gastroenteritis including diarrhea and vomiting that persisted for 1–2 days (Table 1). Six adults were symptomatic. Subjects CHA and CHB reported symptoms only. CH02–05 provided blood samples and CH02 provided a stool sample 2 days after symptom onset. Viral RNA was extracted, reverse-transcribed, and the complementary DNA (cDNA) was screened by reverse-transcription polymerase chain reaction (PCR) with GII human norovirus capsid primers. The resulting GII capsid PCR product was cloned into Topo-XL (Invitrogen, Carlsbad, CA), and subclones were sequenced. The amino acid sequence of the consensus capsid named GII.2 CH was >99.5% identical to numerous GII.2 strain capsids circulating globally in 2016–2017. All 4 sample donors, blood types A, B, or AB, phenotyped as secretor-negative, Lewis-positive, based on the absence of detection of H, A, or B antigens and the presence of Lewis a in salivary samples (Figure 1*A*).

**Table 1:** Demographics of the Familial Norovirus Outbreak Cohort

Outbreak subject	Age, y	Sex	Symptom onset date	Vomit/diarrhea	Fecal sample, day^a^	Serum sample, day^a^	PBMC sample, day^a^	Blood type^b^	Secretor	Lewis
CH02	62	M	11/19/17	–/+	2	8, 30, 180	8, 30, 180	A+	–	+
CH03	55	F	11/19/17	+/+	N/A	8, 30, 180	8, 30, 180	B+	–	+
CH04	20	M	11/19/17	+/+	N/A	8, 30, 180	30, 180	AB-	–	+
CH05	25	M	11/24/17	+/+	N/A	9, 25	9, 25	AB+	–	+
CHA	30	F	11/19/17	+/+	N/A	N/A	N/A	N/A	N/A	N/A
CHB	30	M	11/19/17	+/+	N/A	N/A	N/A	N/A	N/A	N/A

N/A, not available.

a Days after symptom onset.

b Self-reported, as determined by the D’Adamo Personalized Nutrition Home Blood Type Testing Kit (Norwalk, CT).

Cellular immunity after GII.2 infection has been studied in only a single nonsecretor challenged with high-dose virus.^16^ To study cellular and humoral immune responses in this family cluster, we developed a GII.2 CH VLP to probe immune outcomes in this understudied population. The GII.2 capsid gene sequence isolated from donor stool was cloned into the Venezuelan equine encephalitis replicon system and GII.2 CH VLP was produced using previously described methods (Figure 1*B*).^42^^,^^43^ GII.2 CH VLP showed entry-ligand binding profiles consistent with other time-ordered GII.2 strains, notably, robust binding to human type B saliva, weak binding to type A saliva and no binding to pig gastric mucin III (PGM) or other tested ligands.^25^ GII.2 CH did not bind to nonsecretor saliva, incongruous with GII.2 strain infection in the nonsecretor cohort (Figure 1*C*). Donors had detectable blockade antibody titers to GII.2 CH on day 8 that persisted until the end of sample collection (1–6 mo) (Figure 1*D*), consistent with a secondary antibody response derived from memory B cells elicited by previous exposure to a similar virus. CH04 had a delayed peak in GII.2 titers on day 30. The magnitude of the peak response also was lower than observed in the other 3 donors, indicating that GII.2 infection in CH04 may have been a primary infection. These data indicate that all 4 nonsecretors were infected with GII.2 CH, confirming that natural GII.2 infection is secretor-independent.^16^^,^^17^

### Human Norovirus Infection Activates Innate and Adaptive Immune Cells

**Figure 2:**
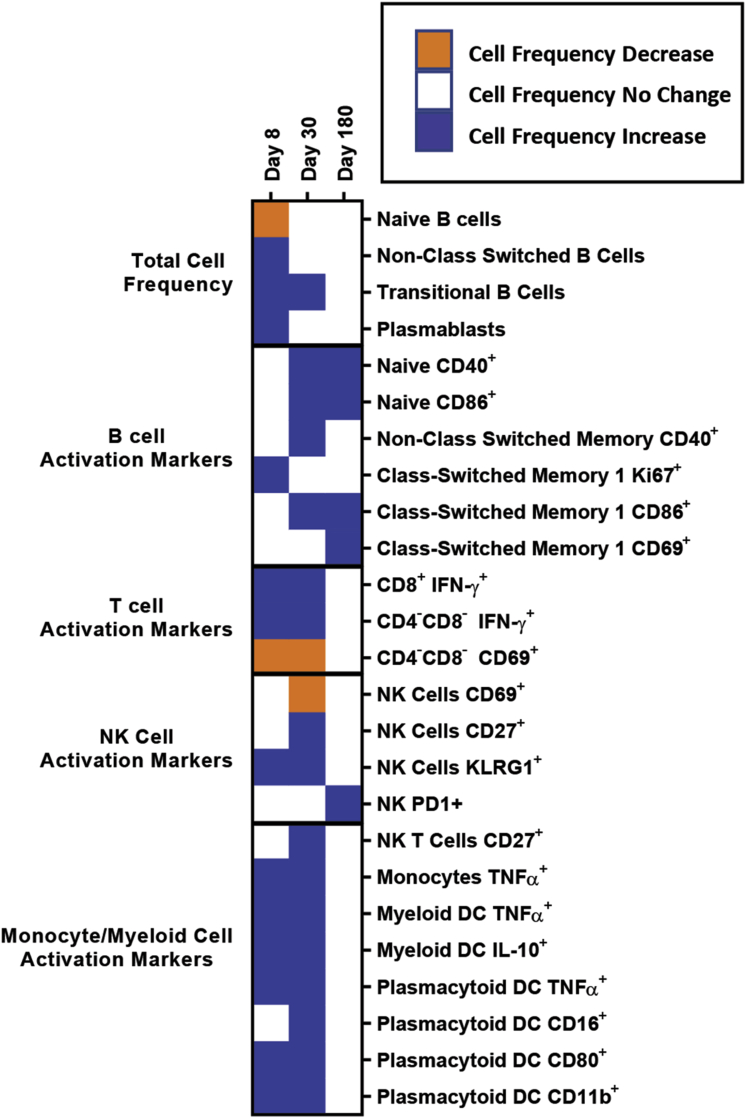
**GII.2 infection broadly activates innate and adaptive immune responses.** PBMCs collected from GII.2 CH–infected donors on days 8, 30, and 180 after infection and 7 blood bank donors were stained for activation markers and the percentage frequency of cells compared at each time point and against the basal response in the blood donors. Cellular subsets different from the donor responses are shown in the heat map (blue indicates an increase, orange indicates a decrease [*P* < .05], and white indicates no change [*P* ≥ .05]). One-way analysis of variance with the Kruskal–Wallis multiple comparison test. DC, dendritic cell.

**Figure 3:**
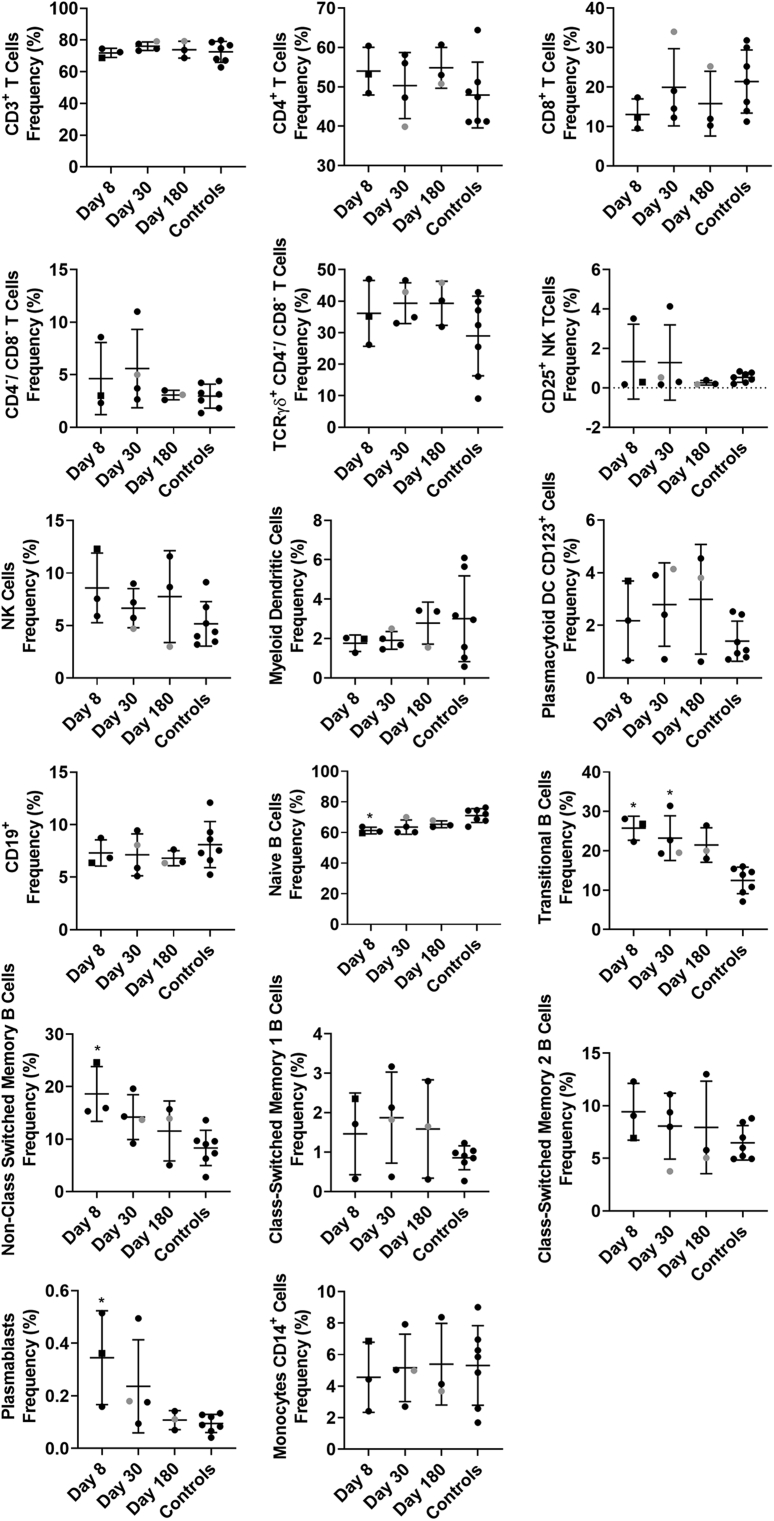
**Phenotypic frequencies of cell populations.** Flow cytometry analysis first removed debris, multiplet events, and dead cells, before measuring lymphocyte and myeloid lineage cell frequencies. Using 3 distinct staining panels, the total T-cell (CD3, plus CD4^+^, CD8^+^, or CD4^-^CD8^-^), NK cell (CD3^-^CD16^+^), NK T-cell (CD3^+^CD16^+^), total B-cell (CD19^+^CD20^+^), monocyte (CD14^+^HLA-DR^+^), myeloid dendritic cell (CD11c^+^HLA-DR^+^CD14^-^), and plasmacytoid dendritic cell (CD123^+^CD14^-^) frequencies of total CD45^+^, live, singlet mononuclear events are shown. The CD4^+^ T, CD8^+^ T, and CD4^-^CD8^-^ T-cell subsets are reported as a frequency of the total CD3^+^ T cells, and the TCRγδ^+^ T cells as a frequency of all CD3^+^CD4^-^CD8^-^ cells. B cells first were classified using CD19 and CD20 coexpression (except plasmablasts) before further phenotyping as naïve (IgD^+^CD27^-^), transitional (CD24^+^CD38^+^), non–class-switched memory (IgD^+^CD27^+^), class-switched memory I (IgD^-^IgM^+^CD27^+^), class-switched memory II (IgD^-^IgM^-^CD27^+^), or plasmablasts (IgD^-^IgM^-^CD27^+^CD38^+^). Means with SD are shown. ∗*P* < .05 compared with healthy donor controls. Donor CH04 data are colored grey for comparison with the other donors. CH04 may be naïve for GII.2 infection, while the other donors likely are not naïve.

**Figure 4:**
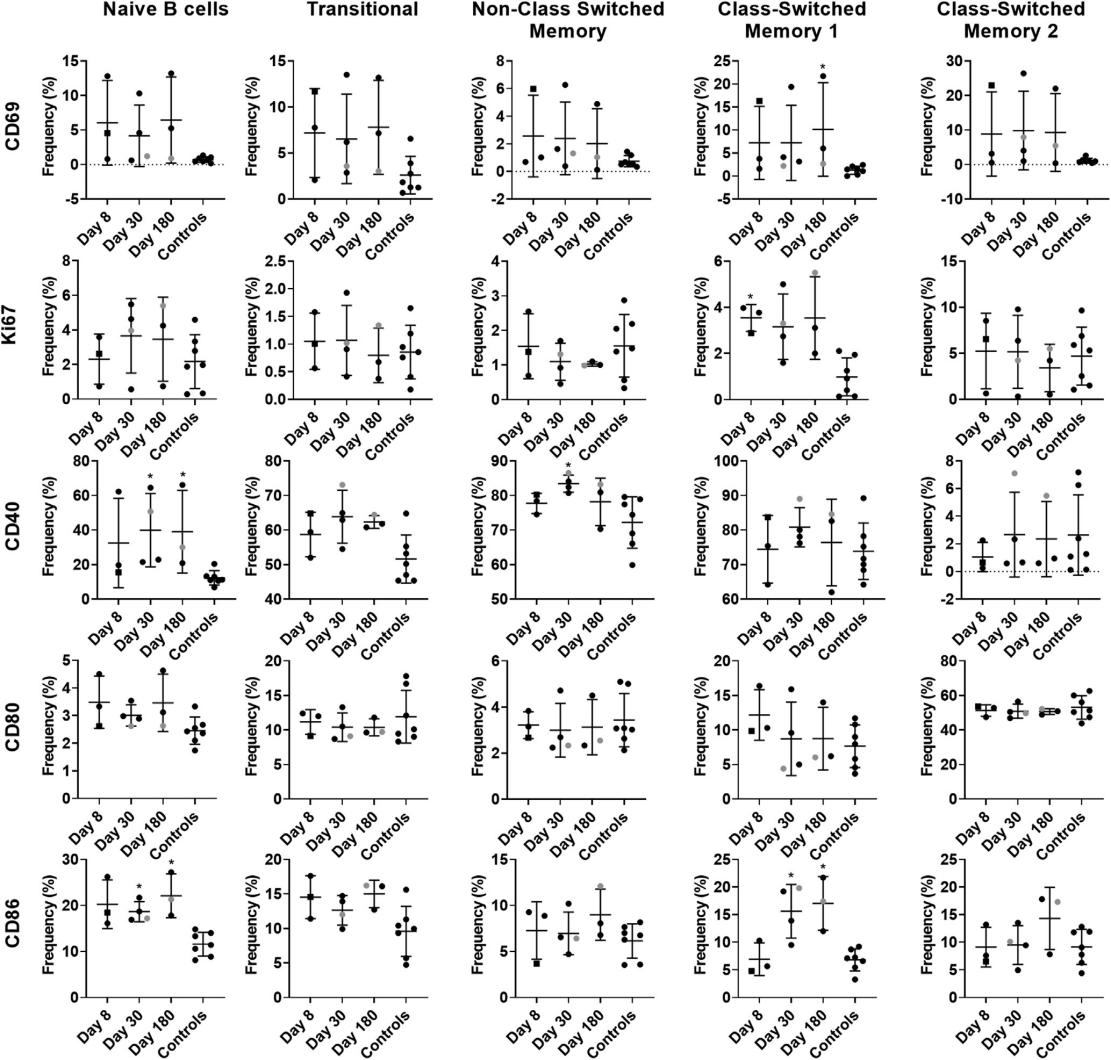
**Activation marker expression patterns in the B-cell compartment.** Unstimulated PBMCs were evaluated for markers of immune modulation in B cells. Cells first were classified as B-cell lineage using CD19 and CD20 coexpression, before further phenotyping as naïve (IgD^+^CD27^-^), transitional (CD24^+^CD38^+^), non–class-switched memory (IgD^+^CD27^+^), class-switched memory I (IgD^-^IgM^+^CD27^+^), or class-switched memory II (IgD^-^IgM^-^CD27^+^). Means with SD are shown. ∗*P* < .05 compared with healthy donor controls. Donor CH04 data are colored grey for comparison with the other donors. CH04 may be naïve for GII.2 infection, while the other donors likely are not naïve.

**Figure 5:**
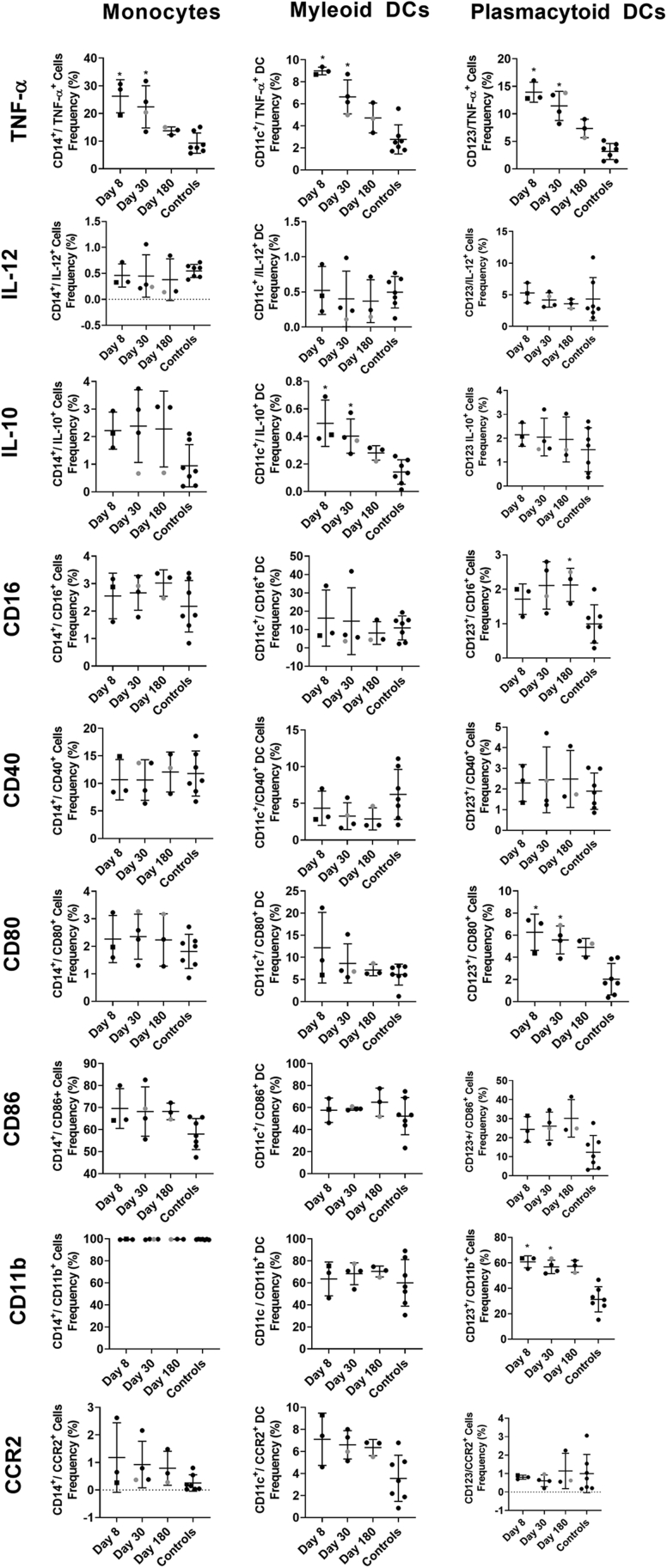
**Activation marker expression patterns within myeloid and dendritic cell (DC) populations.** Unstimulated PBMCs were characterized for expression of activation markers, costimulatory molecules, and cytokines after phenotyping monocytes (CD14^+^HLA-DR^+^), myeloid DCs (CD11c^+^HLA-DR^+^CD14^-^), and plasmacytoid DCs (CD123^+^CD14^-^). Means with SD are shown. ∗*P* < .05 compared with healthy donor controls. Donor CH04 data are colored grey for comparison with the other donors. CH04 may be naïve for GII.2 infection, while the other donors likely are not naïve. CCR, C-C chemokine receptor type 2.

**Figure 6:**
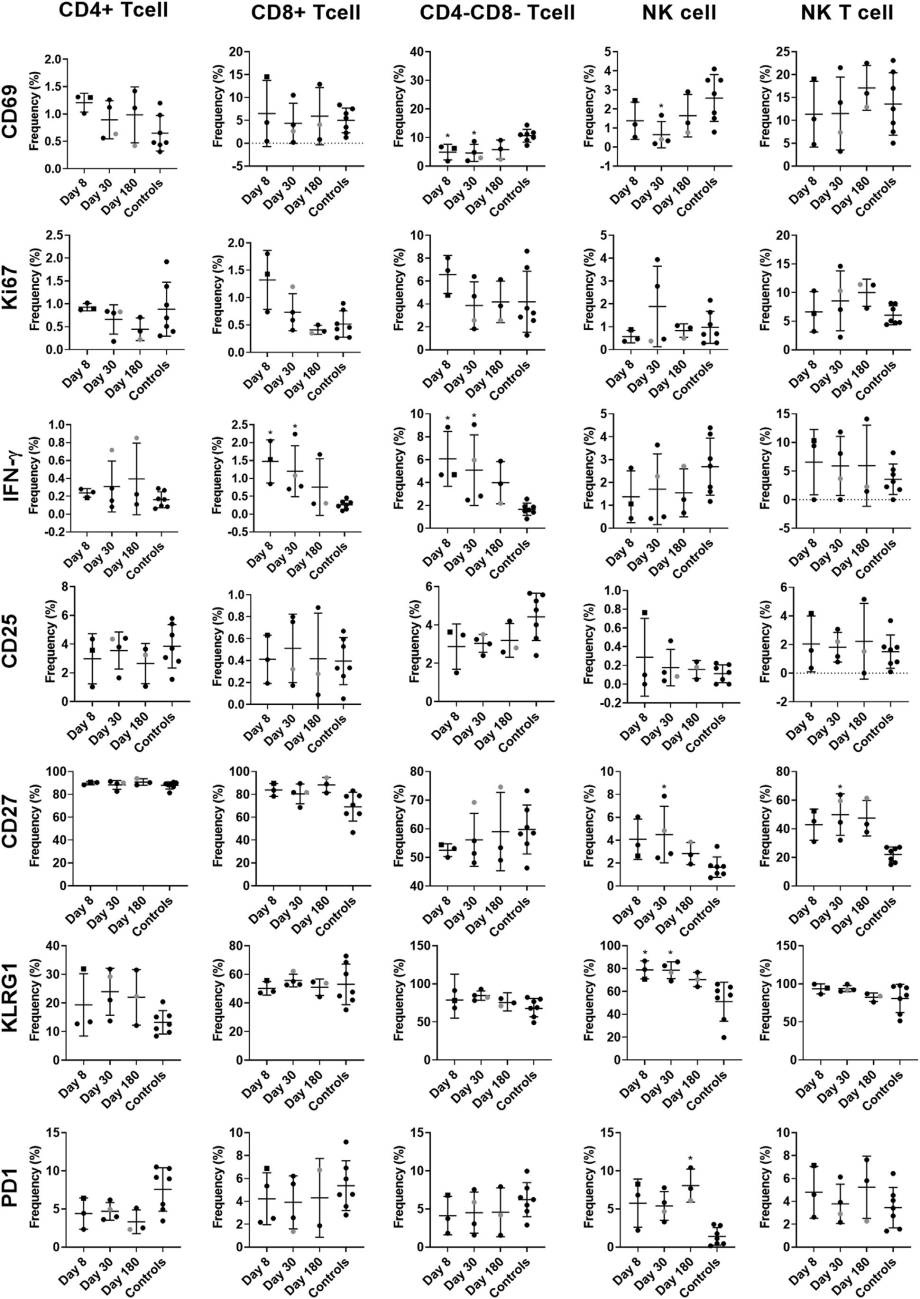
**Activation marker expression patterns in lymphoid cells.** Unstimulated PBMCs were evaluated for markers of immune modulation in CD4^+^ T cells (CD3^+^CD4^+^CD8^-^), CD8^+^ T cells (CD3^+^CD4^-^CD8^+^), double-negative T cells (CD3^+^CD4^-^CD8^-^), NK cells (CD3^-^CD16^+^), and NK T cells (CD3^+^CD16^+^CD4^-^CD8^-^). Means with SD are shown. ∗*P* < .05 compared with healthy donor controls. Donor CH04 data are colored grey for comparison with the other donors. CH04 may be naïve for GII.2 infection, while the other donors likely are not naïve. KLRG, killer cell lectin-like receptor subfamily G member 1; PD1, programmed cell death 1.

To study immune cell activation after GII.2 infection without exogenous VLP stimulation, we used a comprehensive panel of cellular activation and cytokine antibodies, we quantified changes in the immune profile of the GII.2-infected donors with peripheral blood mononuclear cell (PBMC) samples to evaluate the levels of immune activation directly ex vivo (Figure 2, Figure 3, Figure 4, Figure 5, Figure 6)*.* Data from unstimulated samples are different from VLP stimulation experiments in that they report expression patterns present at the time of blood collection. In comparison, VLP stimulation reports the ability of immune cells to specifically respond to VLP, effectively measuring the memory recall response to norovirus. Because pre-infection PBMCs were not available, we included PBMCs from 7 blood donors as a control group to evaluate the relative magnitudes of the responses in our nonsecretor cohort compared with the general population. Age, sex, secretor status, and prior norovirus exposure history were not available for these controls. On day 8 after infection, the frequency of naïve B cells was lower in the infected donors than in control donors, while non–class-switched memory, transitional, and plasmablast B cells all were increased (Figure 2, Figure 3, Figure 4). On day 30, more naïve and memory B cells were positive for activation markers compared with healthy donors, supporting robust antibody production in the infected group, as shown in Figure 1*D*. Activated B-cell populations still were detected on day 180; however, it was unlikely that B-cell activation in response to GII.2 infection persisted for 6 months. Donors were not isolated throughout the study period. Subsequent norovirus or other pathogen exposures during this time are unknown and may have contributed to activated B-cell populations on day 180. Activated IFN-ɣ^+^ T cells, natural killer (NK) cells, tumor necrosis factor (TNF)-α^+^ monocytes, IL10^+^ and TNFα^+^ myeloid dendritic cells, and TNF-α^+^ plasmacytoid dendritic cells persisted at least until day 30 after infection (Figures 2, 3, 5, and 6). Surveying multiple T-cell subtypes (CD4^-^CD8^-^, CD4^+^, and CD8^+^) identified IFN-ɣ secretion in CD4^-^CD8^-^ and CD8^+^, but not CD4^+^, T cells on days 8 and 30. These findings are surprising because CD4^+^ cells have been shown to produce IFN-ɣ after VLP stimulation.^16^^,^^33^ It is unknown if a lack of activation of IFN-ɣ^+^ CD4^+^ cells is a feature of nonsecretor individuals, low sample number, or another mechanism. These data indicate that GII.2 infection activates both innate and adaptive immunity in these donors, resulting in a typical antiviral cytokine production profile^44^ comprising helper T cell (Th)_1_ and Th_2_ cytokines, and highlights the prolonged duration of the cellular immune response, lasting at least 30 days, long after symptom resolution.

### GII.2 Infection Induces Cross-Reactive Cellular and Serologic Immune Responses to Conserved GII Capsid Epitopes

**Figure 7:**
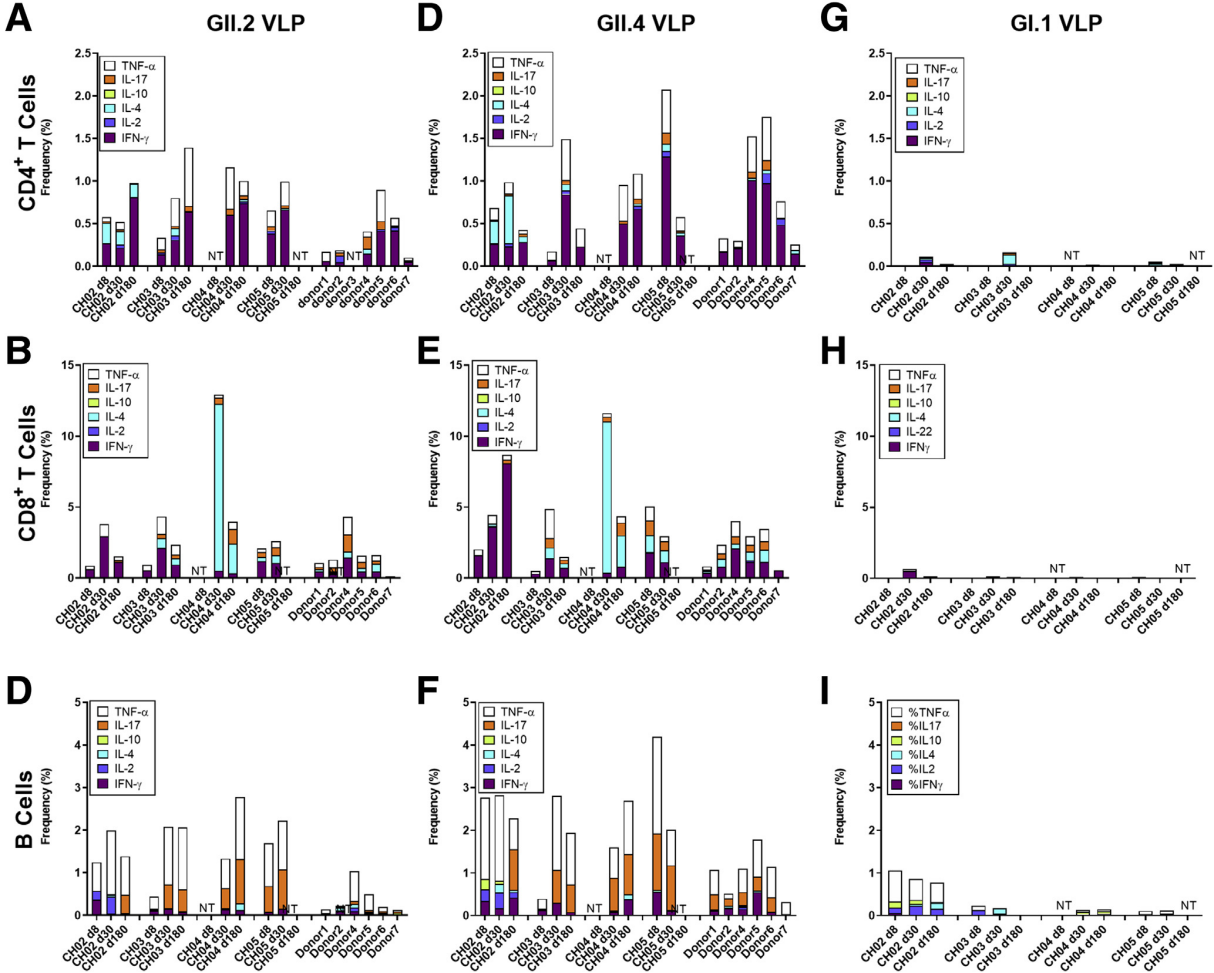
**GII.2 infection induces cross-GII antigen-specific cellular immune responses.** PBMCs from GII.2-infected or control donors were stimulated with 5 μg/mL (*A–C*) GII.2 CH VLP, (*D–F*) GII.4 2012 Sydney VLP, or (*G–I*) GI.1 VLP incubated in the presence of Golgi transport inhibitors, and stained for CD4, CD8, and CD19 and secreted cytokines. Antigen-specific responses in T- and B-cell lymphocytes first were normalized to background frequencies in media control samples, and only the net change over background is reported. In these secretor-negative subjects, CD4^+^ and CD8^+^ T cells predominantly produced IFN-γ (purple), TNF-α (white), and IL4 (light blue) in response to GII.2 or GII.4 human norovirus VLP stimulation. CD19^+^ B cells primarily produced TNF-α (white) and IL17 (orange). One-way analysis of variance with the Kruskal–Wallis multiple comparison test. NT, not tested.

**Figure 8:**
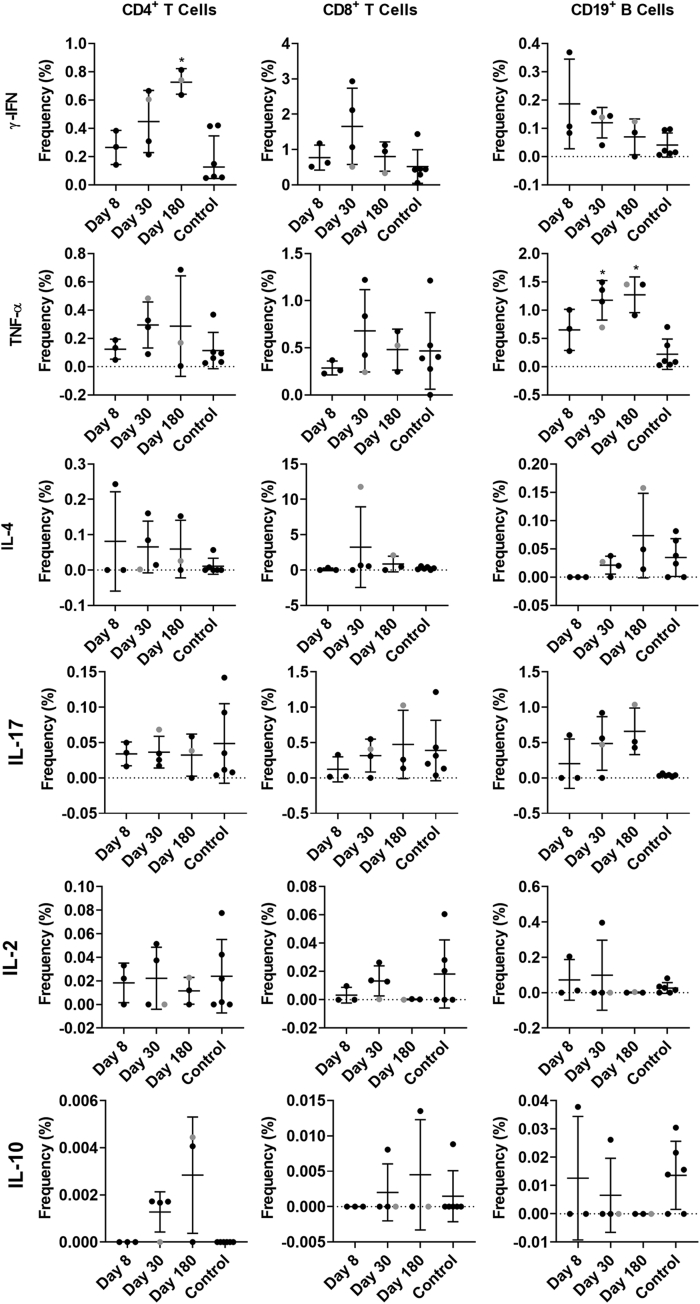
**Cytokine production in response to ex vivo GII.2 VLP stimulation.** PBMCs from GII.2-infected or control donors were stimulated with 5 μg/mL of GII.2 CH VLP, incubated in the presence of Golgi transport inhibitors, and stained for phenotypic cell markers CD3, CD4, CD8, and CD19, and secreted cytokines. Antigen-specific responses in T- and B-cell lymphocytes were first normalized to background frequencies in media control samples, and only the net change over background is reported. Means with SDs are shown. ∗*P* < .05 compared with 6 donor controls. Donor CH04 data are colored grey for comparison with the other donors. CH04 may be naïve for GII.2 infection, while the other donors likely are not naïve. The *dashed line* denotes no frequency detected.

**Figure 9:**
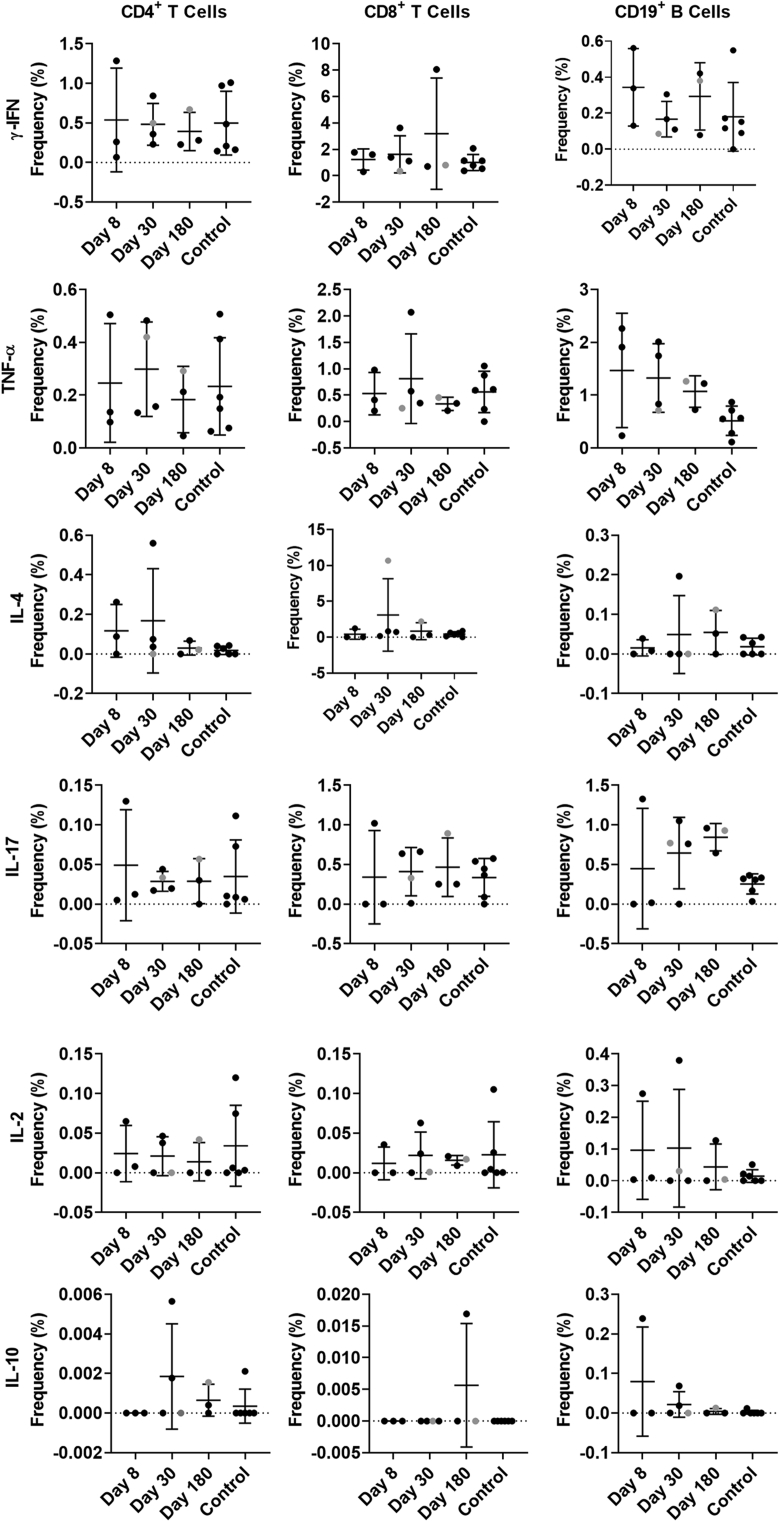
**Cytokine production in response to ex vivo GII.4 VLP stimulation.** PBMCs from GII.2-infected or control donors were stimulated with 5 μg/mL of GII.4 2012 VLPs, incubated in the presence of Golgi transport inhibitors, and stained for phenotypic cell markers CD3, CD4, CD8, and CD19, and secreted cytokines. Antigen-specific responses in T- and B-cell lymphocytes were first normalized to background frequencies in media control samples, and only the net change over background is reported. Means with SD are shown. ∗*P* < .05 compared with 6 donor controls. The *dashed line* denotes no frequency detected.

**Figure 10:**
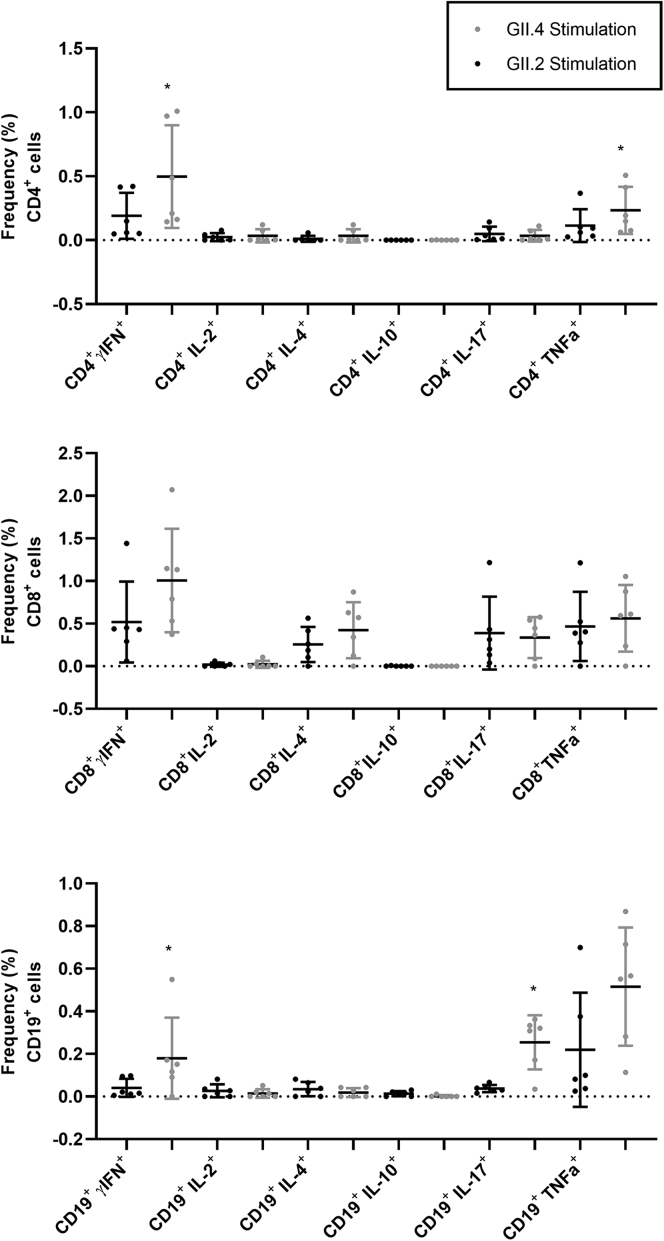
**PBMCs from control donors are more responsive to GII.4 2012 VLP ex vivo stimulation than GII.2 CH stimulation.** PBMCs from control donors were stimulated with 5 μg/mL of GII.4 2012 or GII.2 CH VLP, incubated in the presence of Golgi transport inhibitors, and stained for phenotypic cell markers CD3, CD4, CD8, and CD19, and secreted cytokines. Antigen-specific responses in T- and B-cell lymphocytes were first normalized to background frequencies in media control samples, and only the net change over background is reported. The frequency of cytokine-producing cells after GII.4 2012 and GII.2 stimulation was compared. Means with SDs are shown. ∗*P* < .05 compared with GII.2 VLP response.

Little is known about antigen-specific cellular responses in nonsecretors after norovirus infection. Here, we stimulated available PBMCs (Table 1) from the GII.2-infected familial cohort or blood bank donor controls with either GII.2 CH, GII.4 2012 Sydney, or GI.1 VLP and measured the capsid-specific cytokine responses in T and B cells (Figure 7, Figure 8, Figure 9, Figure 10). After GII.2 VLP stimulation, CD4^+^ and CD8^+^ T cells in both infected and healthy donors responded primarily by producing IFN-γ, TNF-α, and IL4, indicating a Th_1/2_ balanced response to human norovirus infection (Figures 7*A–C* and 8). Cytokine-producing cell frequencies were not different between the 2 groups except CD4^+^ T cells collected on day 180 after infection increased IFN-γ expression compared with control CD4^+^ T cells (Figure 8). The GII.2 and GII.4 VLP-specific CD8^+^ T-cell responses observed in subject CH04 were dominated by high-expression frequencies of IL4, a cytokine that helps drive the differentiation of naïve T cells toward a Th_2_ lineage, proliferation of lymphocytes, B-cell class-switching, and the development of humoral and mucosal immune responses, all of which would be expected during a primary viral infection of the intestinal mucosa. CH04 also showed trends of high CD8^+^ T-cell frequencies, consistent with possible clonal expansion of antiviral, antigen-specific, CD8^+^ T cells (cytotoxic lymphocytes). The cellular immune profile of subject CH04, coupled with the lower serum antibody concentrations, indicate that CH04 may have experienced a primary GII.2 infection during the window of this study. A day 8 sample was not available for this donor and no other control or infected donors had naïve responses, limiting the interpretation of these data.

CD19^+^ B cells primarily stained positive for TNF-α and IL17 in the infected donors, both proinflammatory cytokines produced in response to cellular activation. On days 30 and 180, CD19+ cells expressing TNF-α were more frequent in the infected donors than in healthy controls (Figures 8 and 9). Antigen-specific cellular cytokine response patterns were similar between GII.2 VLP and GII.4 VLP in the GII.2-infected donors, supporting the likelihood of common epitopes between the GII strains. Further supporting shared GII cellular epitopes, infected donors and control donors responded similarly to GII.2 stimulation. However, frequencies of CD4^+^ T cells expressing IFN-γ or TNF-α, and CD19^+^ B cells expressing TNF-α or IL17 in response to VLP stimulation was higher for GII.4 than GII.2 in the healthy donors, likely indicating pre-exposure to GII.4 strains (Figure 10). CD4^+^ and CD8^+^ T cells from study subjects did not produce cytokines in response to GI.1 stimulation, consistent with nonsecretor resistance to GI.1 infection^13^ (Figure 7*G–I*). However, subject CH02 CD19^+^ B cells showed a moderate response to GI.1 stimulation, consistent with cross-GI antibody epitopes in adults (Figure 3*I*).^33^

**Figure 11:**
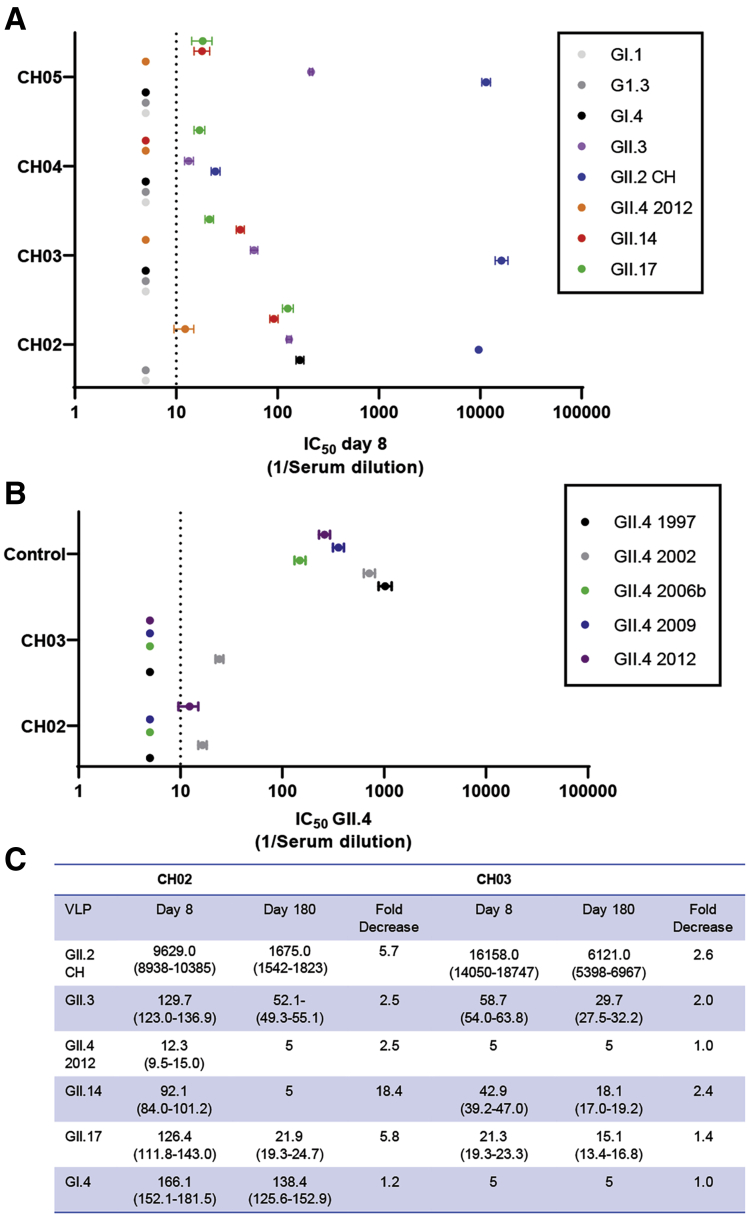
**GII.2 infection induces cross-GII antibody responses in nonsecretors.** (*A*) On day 8, ligand-binding blockade antibodies to GII.2 were greater than titers to other strains, supporting GII.2 infection in all donors. Blockade antibody titers included GII.17 and GII.4 2012, strains associated with infection of secretors. (*B*) Additional strain-specific GII.4 ligand-binding antibody was evaluated on day 8 in CH02 and CH03 and a control donor. (*A* and *B*) Markers denote the means and 95% CIs from a minimum of 2 replicates tested in 2 independent experiments. The *dashed line* is the lower limit of detection. (*C*) Between day 8 and day 180, titers to GII.2, GII.3, GII.14, and GII.17 decreased between 49% and 95%. In comparison, titer to GI.4 in CH02 was relatively stable at a 17% decrease (1.2-fold) change. IC_50_, 50% inhibitory concentration.

All donors had serologic evidence of GII norovirus pre-exposure history as measured by day 8 GII ligand-binding blockade antibody responses. In the donors with a memory antibody response to GII.2 CH, on day 8, titers to GII.2 CH were from ∼50- to 300-fold higher compared with the other tested GII VLPs with titer (Figure 11*A*). The breadth of the serum blockade antibody profiles was consistent between family members. Although titers were low compared with GII.2 CH, on day 8, all 4 subjects had measurable titer to GII.3, GII.14, and GII.17. CH02 also had detectable blockade antibody titer to GI.4 (Figure 11*A*), supporting the CD19^+^ B-cell response to GI.1 in this donor (Figure 7) and the possibility for common blockade antibody epitopes between GI strains.^33^

GII.4 infection in nonsecretors is rare, but not unreported.^11^^,^^45^ To investigate potential GII.4 exposure in the cohort, we screened day 8 sera from the 2 oldest subjects who had the highest day 8 antibody titers (CH02 and CH03) for blockade antibody titer to a panel of pandemic GII.4 strains ranging from 1997 through 2012 (Figure 11*B*). Neither subject had detectable blockade antibody to GII.4 1997, 2006b, or 2009. Both subjects had low but detectable blockade antibody titer to GII.4 2002 and 1 to GII.4 2012, titers were near the limit of detection and lower than titers to GII.3, GII.14, and GII.17, and lower then GII.4 titers in a healthy donor (Figure 11*A* and *B*). Lack of antibody titer to the previous GII.4 pandemic strains suggests the low level of GII.4 blockade antibody detected was cross-reactive antibody generated in response to the current GII.2 infection, because recent GII.4 infections back-boost blockade antibody to ancestral GII.4 strains.^29^^,^^31^ Supporting this hypothesis, the breadth of blockade antibody response on days 8 and 180 in CH02 and CH03 was compared. Titer to GII.2 decreased 83% and 61% between days 8 and 180. Similarly, the titer to GII.3, GII.14, GII.4 2012, and GII.17 decreased 49%–95% between days 8 and 180 as well. In comparison, titer to GI.4 in CH02 was relatively stable during the 180 days of follow-up evaluation at 1.2-fold change (17% decrease) (Figure 11*C*). Together, the cellular and serologic data support the hypothesis that GII.2 infection activates pre-existing GII memory B cells and T cells via common GII epitopes that could be exploited as vaccine or drug targets.

### Bile Salts Are Required for GII.2 CH Binding In Vitro

**Figure 12:**
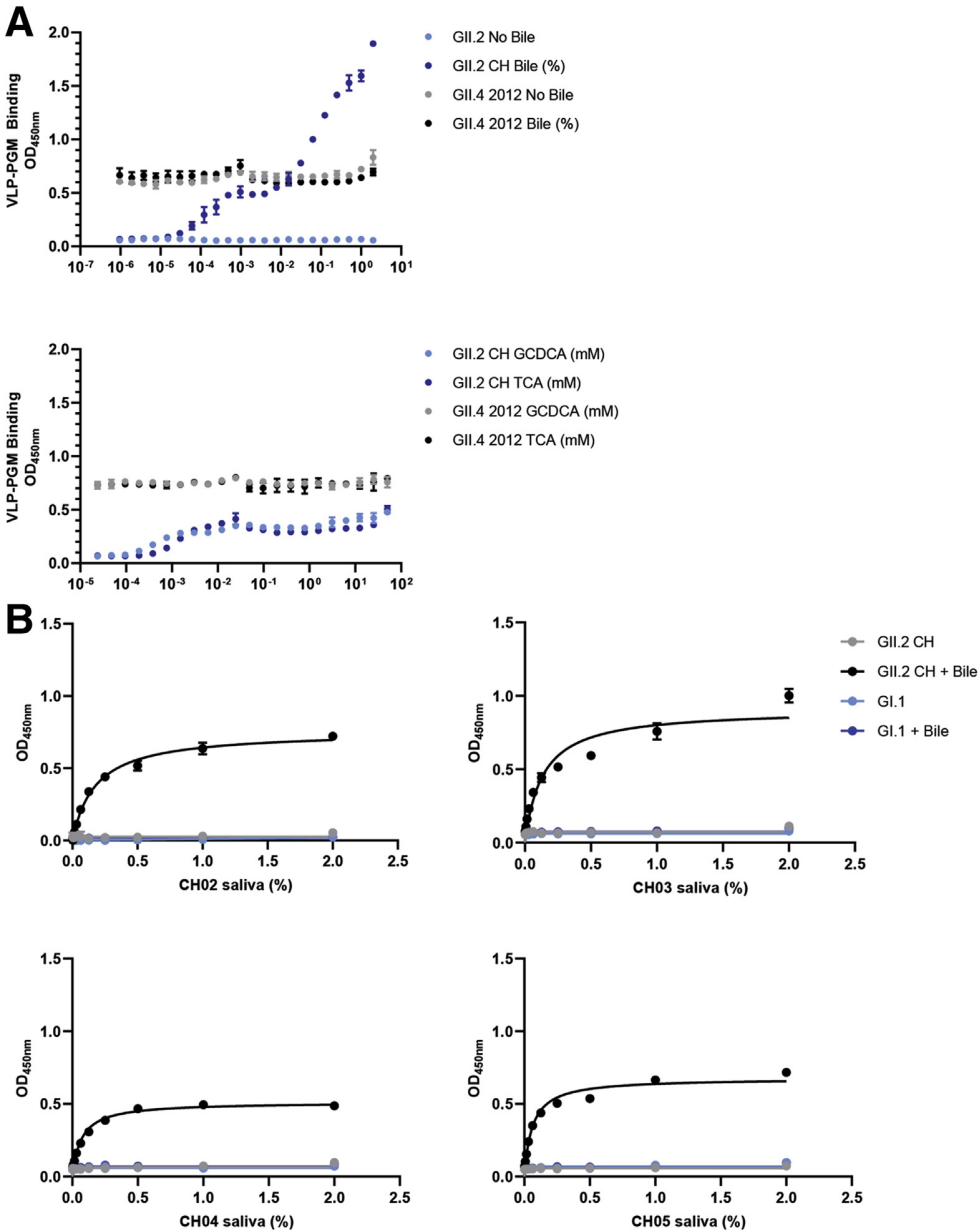
**Bile salts enhance GII.2 CH VLP binding to nonsecretor ligands.** (*A*) GII.2 CH and GII.4 2012 VLP binding to PGM in the absence (grey, light blue) or presence (black, dark blue) of increasing concentrations of crude bovine bile. Bile facilitates GII.2 binding to PGM. Inclusion of GCDCA or TCA purified bile salts had a modest impact on GII.2 CH and no impact on GII.4 2012 VLP binding to PGM. (*B*) GII.2 CH VLPs bind to infected donor salivary ligands in the presence of 1% bile. GI.1 VLP binding to secretor-negative saliva is not affected by bile. Markers denote the means and SDs of at least 1 representative of 2 independent experiments.

**Figure 13:**
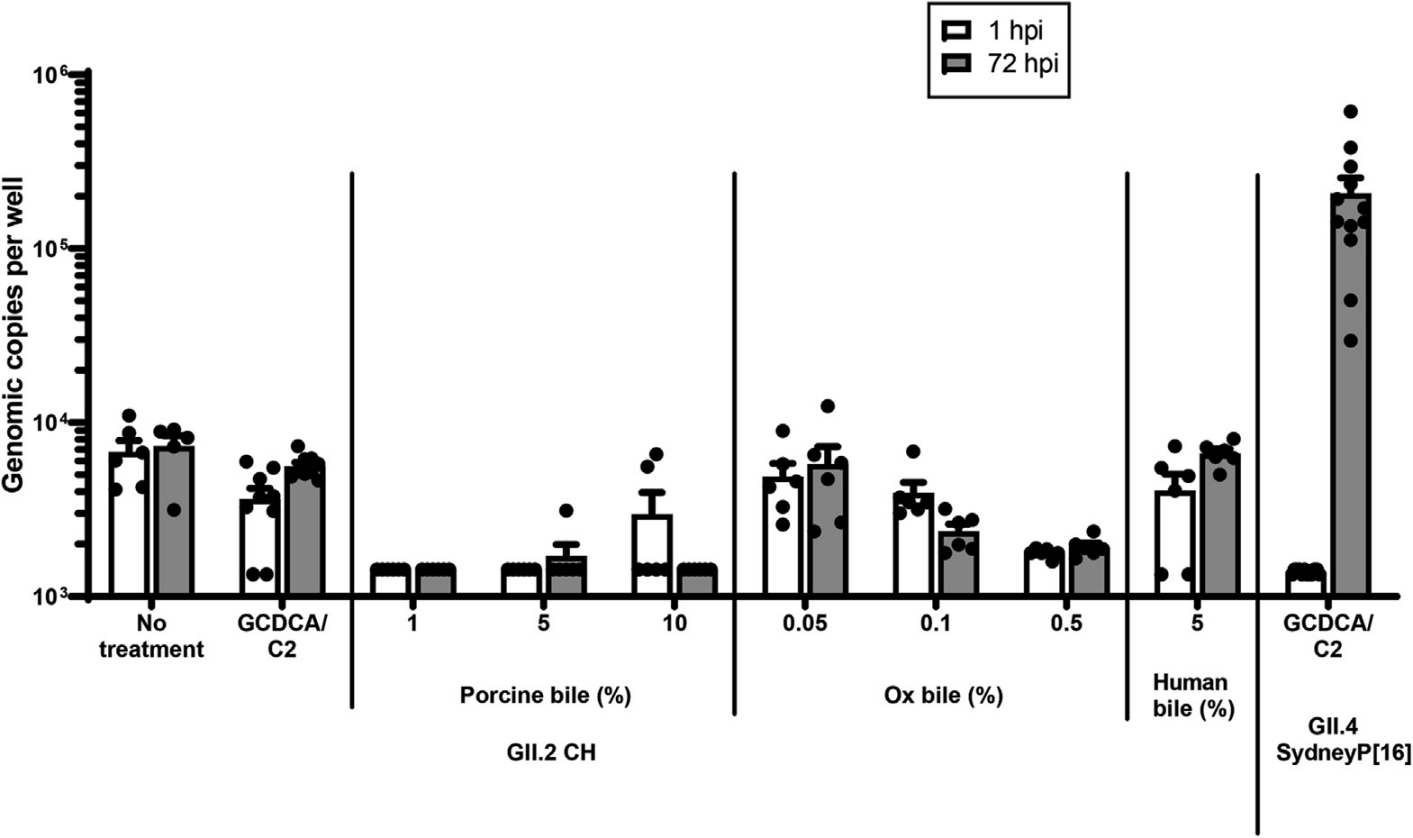
**Bile salts do not support or enhance replication of GII.2 CH in HIE.** Secretor-positive jejunal HIE monolayers (line J2) were pretreated 48 hours before inoculation with GCDCA/C2, pig bile, ox bile, or human bile. Monolayers were inoculated with GII.2 CH at 4.3 × 10^5^ RNA copies/well and incubated for 72 hours. Jejunal HIE monolayers (line J2) also were inoculated with GII.4 Sydney[P16] at 2.1 × 10^5^ RNA copies/well as a positive control for infection. Data represent the means with SDs of 2 experiments with 3 technical replicates for each experiment. hpi, hours postinfection; P16, Polymerase type 16.

GII.2 CH VLPs did not bind to ligands found in the saliva from a nonsecretor donor (Figure 1*C*), posing the question of how does GII.2 infect nonsecretors? Recent reports have indicated that bile enhances growth and/or ligand binding of some human norovirus strains.^27^^,^^46^^,^^47^ Inclusion of bovine bile, but not secondary bile acids glycochenodeoxycholic acid (GCDCA) or taurocholic acid (TCA), conferred dose-dependent binding of GII.2 CH to PGM (Figure 12*A*). Bovine bile, GCDCA, and TCA had no effect on GII.4 2012 Sydney binding to PGM. Strikingly, the addition of bile enabled GII.2 CH VLP to bind to saliva from the 4 nonsecretor donors (Figure 12*B*). As expected, bile did not enable binding of GI.1 VLP to the saliva of nonsecretors, who are genetically resistant to GI.1 infection.^13^ Inclusion of bovine bile, porcine bile, human bile, or GCDCA/C2 did not support or enhance GII.2 CH growth in vitro (Figure 13). Many natural isolates of human norovirus do not replicate in vitro, especially those isolated from adults.^48^ These data not only support a complex role for bile in human norovirus infection and replication but argue that 1 or more unknown constituents in bile play a critical role in the GII.2 lifecycle.

## Discussion

HBGA expression mediates infection and shapes immunity to a diverse set of human enteric pathogens, including norovirus, rotavirus, cholera, *Escherichia coli*, and *Helicobacter pylori*, and others.^49^^,^^50^ Global populations range from 30% to 70% HBGA nonsecretors. Because genetically resistant individuals either may not respond to or respond differently to a human norovirus vaccine, the impact of host susceptibility polymorphisms on immune performance and vaccine outcomes remains understudied. Notably, rotavirus vaccination efficacy rates range between 40% and 90% globally.^51^ Nonsecretors are genetically resistant to some prevalent rotavirus strains, shed less virus after vaccination, and experience fewer vaccine failures.^52^, ^53^, ^54^, ^55^ These data show that HBGA expression patterns are a driving force in vaccine efficacy for enteric pathogens, especially those vaccines that use VLP or live attenuated vaccines that bind HBGA attachment factors for entry.

GII.2 infection of secretors may occur via direct binding of the virus to select secretor HBGAs. Our data clearly show natural GII.2 infection of a nonsecretor population. Moreover, we show that bile acids may override the genetic advantage of less-diverse HBGA expression in nonsecretors by improving the avidity of GII.2 binding to nonsecretor HBGAs, potentially paving the way for infection. These data are supported by evidence of bile-enhanced growth of GII.2, GII.3, GII.17, and GI.1 human norovirus in vitro and GII.1 VLP in vitro binding to natural carbohydrate ligands.^27^^,^^47^^,^^48^ Inclusion of bile was not sufficient to gain in vitro replication of the GII.2 CH virus in secretor human intestinal enteroid (HIE) cells, emphasizing our incomplete understanding of human norovirus cellular entry mechanisms. Failure to infect these cultures may reflect commonly reported virus sampling problems, reflecting the need to test the ability of a spectrum of bile salts to enhance growth of other GII.2 virus samples in cell culture.^48^ Exogenous bile improves GI.1 propagation in the HIE system,^47^ but did not affect binding of GI.1 VLP to the HBGAs in nonsecretor saliva, indicating that bile cannot override genetic resistance. These data articulate the complex interaction patterns of bile and bile types with different human norovirus. Effects are virus strain– and bile type–dependent, and can be at the cellular or virus level, shown by improved virus production in bile-treated HIE cells^47^ and improved VLP binding to ligands in biochemical assays (here and discussed by Kilic et al^27^). Thus, in addition to HBGAs, 1 or more specific components of bile also is likely to be an essential co-factor for human norovirus attachment and infection.

Susceptibility mediates infection, thus HBGA expression patterns on cells also shape immunity. Previously, in a small group of adults immunized with a bivalent human norovirus VLP-based vaccine, secretors and nonsecretors responded similarly in breadth of ligand binding–blocking antibodies to human norovirus strains not included in the vaccine, early after vaccination.^29^ However, only secretors with pre-existing blockade antibody titer maintained long-term blocking antibody breadth,^30^ indicating host genetics and pre-exposure history co-define the breadth and durability of human norovirus immune responses. Similarly, rotavirus vaccination uptake was not different between secretors and nonsecretors, but neither duration nor breadth of antibody responses across strains were evaluated in this study, limiting interpretation of the effect of secretor status on rotavirus vaccine response.^56^ Blockade antibodies correlate with protection from infection and have been confirmed to reflect norovirus neutralizing antibodies accurately.^35^, ^36^, ^37^

There were limitations to this study, including an absence of viral titers, measuring peripheral responses to a mucosal pathogen, and lack of baseline samples in this retrospective study. We included a panel of healthy blood donors as a comparison group in the absence of pre-exposure sample from the donors, however, no demographics or exposure history for these control donors was available and they may not represent baseline samples from the nonsecretor infected donors accurately. Similarly, the small cohort studied here may not reflect nonsecretors as a whole, particularly the single subject who may have been experiencing a primary GII.2 infection. Despite study limitations, the rare nonsecretor family cohort provided an opportunity to study in detail human norovirus immunity in nonsecretors experiencing a natural GII.2 infection. Here, serum blockade antibodies persist at least 180 days after GII.2 infection. For at least 30 days, antibody responses were supported by NK cells, monocytes, dendritic cells, and T cells producing antiviral cytokines IFN-ɣ and TNF-α and anti-inflammatory IL10, in agreement with previous reports.^16^^,^^33^^,^^57^, ^58^, ^59^ Congruently, drastically reduced B-cell numbers coupled with less-responsive T cells contribute to long-term human norovirus infection in immune-compromised populations,^60^ supporting a key role of antibody and adaptive immunity in human norovirus protection.

Many human norovirus challenge/vaccine studies exclude nonsecretors, thus relatively little is known about the breadth of immunity in this population, which likely has a different profile of human norovirus exposure compared with secretors whose immunity is shaped overwhelmingly by recurrent infection with pandemic GII.4 strains. It is not known if nonsecretors have the pre-exposure history to support protection after vaccination with GII.4 immunogen-containing vaccines. Serologic and cellular immune responses in our GII.2-infected cohort support cross-GII immunity in nonsecretors for at least 6 months. GII.2 infection boosted cross-reactive blocking antibodies to GII.3, GII.14, and GII.17, as well as T-cell responses to GII.4, despite the lack of clear serologic evidence of previous GII.4 exposure. CH04, the youngest donor, had the least blockade antibody breadth on day 8, and delayed peak GII.2 antibody responses, indicating this donor likely was experiencing a primary GII.2 infection. Supporting common GII epitopes, CH04 still mounted GII.4 T-cell and GII.3 and GII.17 blockade antibody responses similarly to the other donors. No T-cell responses to GI.1 were detected in any of the tested samples, indicating a lack of cross-genogroup epitopes, as previously reported.^16^ Despite evidence of their existence, no cross-genotype blockade antibody epitopes have been mapped,^16^^,^^29^^,^^33^^,^^61^ although common GII T-cell epitopes have been described.^16^^,^^57^^,^^62^^,^^63^ Serum samples collected here are well positioned to select for antibodies that bind to conserved epitopes by multiple rounds of affinity selection followed by co-crystal structures of recombinant antibodies with viral domains, as previously described by our group for GII.4.^31^ Conserved epitopes likely represent preferred targets for vaccine design and therapeutics, especially given the unpredictable prevalence patterns for the GII human norovirus, because GII.17, GII.6, and GII.2 all have caused excess cases in individual years since 2011.^10^^,^^64^

How different exposure histories between secretors and nonsecretors shape immunity is unknown. As development of human norovirus vaccines progress, studies should include nonsecretors to define immunity in this population and compare individual and group outcomes by HBGA expression. These data may provide valuable information on the antigenic relationship between strains and the duration and breadth of immune responses in the absence of regular GII.4 boosting. To date, all published human norovirus vaccine studies have focused on adults, while the aged and very young populations experience the most severe disease outcomes. In fact, the effect of nonsecretor HBGA expression may be most impactful in young children, the primary intended target for a human norovirus vaccine, in whom pre-exposure history will be limited before vaccination. Findings here indicate that nonsecretors recognize B- and T-cell epitopes shared between secretor-dependent and -independent strains of human norovirus, including GII.4, which may be boosted by vaccination to provide broad immunity to human norovirus, and provide mechanistic support for recent reports of GII.2 protection from infection after GII.4 vaccination of adults (Ivo Sondereggar, PhD, Takeda Vaccine Business Unit, December 18, 2019, personal communication).

## Methods

All authors had access to the study data and reviewed and approved the final manuscript.

### Study Subjects

This study was approved by the University of North Carolina Institutional Review Board (protocol 14-3055). Written informed consent was obtained before participation. No additional samples or patient follow-up studies are available. Cryopreserved PBMCs from healthy donor controls were purchased from Gulf Coast Regional Blood Center (Houston, TX). No demographic data for the control PBMC donors are available, as per deidentification requirements.

### VLP Production

Viral detection, sequencing, cloning, and VLP expression were performed as previously described.^43^^,^^65^ Briefly, the stool sample collected from CH02 at 2 days after symptom onset was diluted to approximately 10% with phosphate-buffered saline, vortexed, and then centrifuged at 3000 × *g* for 10 minutes. RNA was extracted from 250 μL of the clarified supernatant using 750 μL TRIzol LS (Life Technologies, Carlsbad, CA), as recommended. RNA then was isolated using a QIAamp Viral RNA Mini Kit (Qiagen, Germantown, MD) and cDNA was made using 10 μL of RNA and a Superscript^TM^ II RT kit (Invitrogen, Carlsbad, CA), as recommended. The cDNA was digested with 0.01 μg RNase (DNase free; Roche, Indianapolis, IN), purified with a QIAquick PCR Purification Kit (Qiagen), and amplified by PCR using primers targeting open reading frame 2 (5’-ggccacgcgtcgactagtacttttttttttttttttttt-3’, 5’-atgaagatggcgtcgartgacgcc-3’). Provided material was sufficient to sequence capsid genes only. The amplicons were cloned into Tope XL (Invitrogen). GII.2 CH represents the consensus sequence of the amplicons. The GII.2 CH capsid gene was synthesized by Bio-Basic, Inc (Amherst, NY). VLPs were expressed in baby hamster kidney cells (ATCC CCL-10) from Venezuelan equine encephalitis virus replicons expressing human norovirus open reading frame 2, as described previously.^6^^,^^42^^,^^43^ Particle integrity was confirmed by electron microscopy visualization of negative-stained particles.

### VLP-Ligand Binding Assays

VLPs bound to PGM type III (Sigma Aldrich, St. Louis, MO) or human type B saliva were detected by rabbit polyclonal antiserum (Cocalico Biologicals, Stevens, PA), as described previously.^66^^,^^67^ For assays including bile salts, stocks were prepared in PBS, aliquoted, and stored at -20°C, and reagents were purchased from Sigma Aldrich: bovine bile (B3883), GCDCA (G-0759), and TCA (T4009). Data were graphed by single-site binding curve analysis in GraphPad Prism 8.1.1 (San Diego, CA).^68^

### Blockade of VLP–Ligand Binding Assays

Ligand-binding blockade antibody assays were performed at 37°C with 0.25 ug/mL VLP.^32^^,^^67^ VLPs were pretreated with decreasing concentrations of plasma for 1 hour and transferred to PGM or human type B saliva–coated (GII.2 CH VLP only) plates for 1 hour. The percentage of control binding was compared with a no-plasma pretreatment. The mean 50% inhibitory concentration titers and 95% CIs were determined from dose-response sigmoidal curve fits using GraphPad 8.1.1.^29^^,^^30^ Plasma that did not block at least 50% of VLPs binding to binding ligand at the lowest dilution tested were assigned a titer equal to 0.5 times the limit of detection for statistical analysis.

### Secretor Phenotyping

Saliva was heat-treated at 100°C for 10 minutes, centrifuged at 21,000 × *g* for 1 minute, and stored at -20°C until use. High-binding, 96-well, microtiter plates were coated with processed saliva 2-fold serially diluted starting at 2% before the addition of mouse monoclonal antibodies to A and B antigens (1/20 dilution, Ortho-Clinical Diagnostics, Inc, 606209547 and 606209567), Lewis a or Lewis b antigens (1 μg/mL, SC-51512 and SC-51513; Santa Cruz Biotechnologies, Inc, Santa Cruz, CA), respectively. Incubation temperature, washing, and color development were performed as described for the blockade antibody assay. Data were graphed by single-site binding curve analysis in GraphPad Prism 8.1.1.^68^

### Flow Cytometry

Cryopreserved PBMCs from study participants and from healthy donor controls (N = 7; purchased from Gulf Coast Regional Blood Center) were batch-analyzed for flow cytometry. After thawing and washing, cells were suspended in RPMI 1640 media supplemented with 5% fetal bovine serum and 1× antibiotic-antimycotic (Gibco, Gaithersburg, MD) at 2 × 10^6^ cells/mL, and aliquoted in 1-mL culture volumes. For antigen-specific analysis, cultures were treated with purified anti-CD28 and CD49d antibodies (BD Biosciences) plus the following: (1) 5 μg/mL GII.2 CH VLP; (2) 5 μg/mL GII.4 2012 Sydney VLP; (3) 5 μg/mL GI.1 Norwalk VLP; (4) 1× Cell Stimulation Cocktail (phorbol 12-myristate 13-acetate and ionomycin cocktail; eBioscience, San Diego, CA); or (5) media alone. The concentration of VLP for antigenic stimulations was titrated previously, and empirically determined to be 5 μg/mL. Cultures were gently mixed and incubated at 37°C for 1 hour before the addition of 1× Protein Transport Inhibitor (eBioscience). Cultures were incubated for an additional 16 hours before intracellular staining using Fixation/Permeabilization Solution and Perm/Wash (BD Biosciences) according to the manufacturer’s recommendations. In addition to human norovirus-specific responses in lymphocytes, 3 additional staining panels were used to evaluate baseline levels of immune activation (Table 2, Table 3, Table 4, Table 5). Unstimulated PBMCs were aliquoted to additional tubes and stained using titrated antibodies for surface and intracellular markers using the same procedure described earlier. Cells were stained using a Fixable Live/dead Discriminator Dye (ThermoFisher, Waltham, MA) to eliminate dead cells during gating analysis. Fluorescence minus one and unstained cells were assayed in parallel as staining controls. After fixation, cells were transferred to staining buffer and refrigerated overnight until analysis the following day on an LSRII cytometer (BD Biosciences). Cell populations were gated first to exclude debris, doublet/multiplet events by 2 comparisons (FCS-A vs FCS-H and SSC-H vs SSC-W), and dead cells staining positive for the amine-reactive discriminator dye before downstream enumeration of marker expression in FlowJo version 10 software (Treestar, Ashland, OR). All cell frequencies are reported normalized to total live, singlet, CD45+ events, or as the frequency of the parent population, where indicated. Antigen-specific responses in T- and B-cell lymphocytes were first normalized to background frequencies in media control samples, and only the net change over background is reported.

**Table 2:** B-Cell Activation and Memory Flow Cytometry Antibody Panel

Marker	Fluor	Clone	Vendor	Catalog
Live/dead	UV	N/A	Invitrogen	L34962
CD19	AF488	HIB19	Biolegend (San Diego, CA)	302219
CD20	APCFIRE750	2H7	Biolegend	302358
IgD	BV785	IA6-2	Biolegend	348242
IgM	PEDazzle594	MHM-88	Biolegend	314530
CD38	PECy7	HB-7	Biolegend	356608
CD27	PerCPCy5.5	M-T271	Biolegend	356408
CD24	BV605	ML5	Biolegend	311124
CD80	BV650	L307.4	BD (Franklin Lakes, NJ)	564158
CD86	AF700	2331 (FUN-1)	BD	561124
CD69	BV711	FN50	BD	563836
PD-1	BV421	EH12.2H7	Biolegend	329920
CD40	PE	5C3	BD	555589
Ki-67	AF647	Ki-67	Biolegend	350510

N/A, not available; PD-1, Programmed cell death 1.

**Table 3:** T, NK, and NK T-Cell Activation Flow Cytometry Antibody Panel

Marker	Fluor	Clone	Vendor	Catalog
Live/dead	UV	N/A	Invitrogen	L34962
CD3	BB515	UCHT1	BD (Franklin Lakes, NJ)	564465
CD4	BV605	RPA-T4	Biolegend (San Diego, CA)	300556
CD8	AF700	RPA-T8	BD	557945
TCRγδ	PECF594	B1	BD	562511
CD16	AF780	CB16	eBioscience	47-0168-42
CD56	BV786	5.1H11	Biolegend	362550
CD25	BV650	BC96	Biolegend	302634
CD27	PerCPCy5.5	M-T271	Biolegend	356408
CD69	BV711	FN50	BD	563836
PD-1	BV421	EH12.2H7	Biolegend	329920
KLRG1	PE	SA231A2	Biolegend	367712
Ki-67	AF647	Ki-67	Biolegend	350510
IFN-γ	PECy7	4S.B3	eBioscience	25-7319-82

N/A, not available; PD-1, Programmed cell death 1; TCR, T cell receptor.

**Table 4:** Myeloid Cell Activation Flow Cytometry Antibody Panel

Marker	Fluor	Clone	Vendor	Catalog
Live/dead	UV	N/A	Invitrogen	L34962
CD11b	BV510	ICRF44	BD (Franklin Lakes, NJ)	563088
CD11c	BUV661	B-ly6	BD	565067
HLADR	AF488	L243	Biolegend (San Diego, CA)	307656
CD123	PerCPCy5.5	7G3	BD	558714
CD14	BV786	M5E2	BD	563698
CD16	BV421	3G8	BD	562874
CCR2	AF647	48607	BD	558406
CD80	BV650	L307.4	BD	564158
CD86	AF700	2331 (FUN-1)	BD	561124
CD40	BV605	5C3	Biolegend	334336
IL10	PECF594	JES3-19F1	BD	562400
IL12	PE	C15.6	BD	554479
TNF-⍺	PECy7	MAb11	BD	557647

N/A, not available.

**Table 5:** Antigen-Specific Lymphocyte Response Flow Cytometry Antibody Panel

Marker	Fluor	Clone	Vender	Catalog
Live/dead	UV	N/A	Invitrogen	L34962
CD3	BB515	UCHT1	BD (Franklin Lakes, NJ)	564465
CD4	BV605	RPA-T4	Biolegend (San Diego, CA)	300556
CD8	AF700	RPA-T8	BD	557945
CD19	BV650	SJ25C1	BD	563226
CD45RA	APCFIRE750	HI100	Biolegend	304152
CCR7	PerCPCy5.5	150503	BD	561144
IL2	PE	MQ1-17H12	Biolegend	500307
IL4	AF647	8D4-8	Biolegend	500712
IL10	PECF594	JES3-19F1	BD	562400
IL17A	BV786	N49-653	BD	563745
IFN-γ	PECy7	4S.B3	eBioscience	25-7319-82
TNF-⍺	BV421	MAb11	Biolegend	502932

N/A, not available.

### HIE Culture and Inoculation

Secretor-positive jejunal HIE cultures (J2 lines) were grown as undifferentiated 3-dimensional cultures, dissociated into a single-cell suspension, and plated as monolayers as described previously.^47^^,^^48^ All infections were performed in triplicate on 100% confluent 4-day-old differentiated HIE (J2 line) monolayers. In some experiments, monolayers were pretreated 48 hours before inoculation with porcine bile (1%–10%; Sigma), ox bile (0.05%–0.5%; Sigma, St. Louis, MO), human bile (5%), 500 μmol/L GCDCA (Sigma), or GCDCA plus 50 μmol/L ceramide (C2; Santa Cruz Biotechnologies, Inc).

Ten percent stool filtrates from the GII.2 CH norovirus–positive stool and a GII.4 Sydney [Polymerase type 16]–positive stool sample were prepared as described previously.^48^ To determine viral infectivity, duplicate 96-well plates were inoculated with GII.2 CH (4.3 × 10^5^ copies/well) or GII.4 Sydney [Polymerase type 16] (2.1 × 10^5^ copies/well). After a 1-hour incubation at 37°C and 5% CO_2_, monolayers were washed twice with complete media with growth factors and 100 μL differentiation medium containing porcine bile (1%–10%), ox bile (0.05%–0.5%), human bile (5%), or 500 μmol/L GCDCA plus 50 μmol/L C2. For each set of infections, 1 plate was frozen immediately at -70°C and a duplicate plate was incubated at 37°C, 5% CO_2_, for 72 hours and frozen at -70°C. Norovirus RNA from cells and media at 1 hour and 72 hours after infection was extracted using the MagMax-96 Viral RNA Isolation Kit (Applied Biosystems, Foster City, CA) and quantified by reverse-transcription quantitative PCR as described previously.^48^ Experiments were performed twice (3 technical replicates each time) from independent enteroid preparations. Data are presented as means ± SD.

### Statistical Analysis

Statistical analyses were performed using GraphPad Prism 8.1.1.^6^^,^^29^ Ligand maximum binding and dissociation constants were determined by single-site binding curve analysis. The 50% inhibitory concentration values were log transformed for analysis. Antibody titer and binding measurements and response between GII.2 and GII.4 VLP stimulation among controls were compared by an unpaired *t* test with Welch correction (*t* test) or ordinary 1-way analysis of variance with the Dunnett multiple comparison test (analysis of variance). Cell population frequencies and cytokine production after in vitro stimulation between infected donors and controls were compared by 1-way analysis of variance with the Kruskal–Wallis multiple comparison test. A difference was considered significant if *P* < .05.
